# Predicting the Risk of Melanoma Metastasis Using an Immune Risk Score in the Melanoma Cohort

**DOI:** 10.3389/fbioe.2020.00206

**Published:** 2020-03-31

**Authors:** Yang Sheng, Cheng Yanping, Liu Tong, Liu Ning, Liu Yufeng, Liang Geyu

**Affiliations:** ^1^Key Laboratory of Environmental Medicine Engineering, Ministry of Education, School of Public Health, Southeast University, Nanjing, China; ^2^Department of Plastic and Reconstructive Surgery, The Second Hospital of Nanjing, Nanjing University of Chinese Medicine, Nanjing, China

**Keywords:** primary melanoma, metastatic melanoma, metastasis risk, immune risk score, nomogram

## Abstract

Melanoma is a highly aggressive cancer, attracting increasing attention worldwide. The 5-year survival rate of patients with metastatic melanoma is low. Therefore, it is critical to identify potential effective biomarkers for diagnosis of melanoma metastasis. In the present study, the melanoma cohort and immune genes were obtained from the Cancer Genome Atlas (TCGA) database and the ImmPort database, respectively. Then, we constructed the immune risk score (IRS) using univariate and multivariate logistic analysis. The area under the curve (AUC) of IRS in sequencing samples and the initial diagnosis patients was 0.90 and 0.80, respectively. Besides, IRS could add benefits for metastasis diagnosis. For sequencing samples, IRS (OR = 16.35, 95% CI = 8.74–30.59) increased the odds for melanoma metastasis. Similar results were obtained in the initial diagnosis patients (OR = 8.93, 95% CI = 3.53–22.61). A composite nomogram was built based on IRS and clinical information with well-fitted calibration curves. We further used other independent melanoma cohorts from Gene Expression Omnibus (GEO) databases to confirm the reliability and validity of the IRS (AUC > 0.75, OR > 1.04, and *P* value < 0.01 in all cohorts). In conclusion, IRS is significantly associated with melanoma metastasis and can be a novel effective signature for predicting the metastasis risk.

## Introduction

Cutaneous malignant melanoma (hereafter called melanoma), a highly aggressive cancer, only accounts for about 2% of skin cancers but causes the most deaths of skin cancers because of rapid progression and metastasis to regional lymph nodes as well as distant organs (Linares et al., 2015; Tracey and Vij, 2019). According to *cancer statistics 2019*, there will be approximately 95,830 new cases of melanoma *in situ* of the skin and around 3098 cutaneous invasive melanoma at an estimate in the US alone (Siegel et al., 2019). Several studies showed that surgical excision of primary melanoma had a high cure rate (Schadendorf et al., 2018). However, melanoma has the high potential for invasion and metastasis. If melanoma is accompanied by lymph node or distant metastases, it will be life-threatening with only 15–20% 5-year survival rate (Weiss et al., 2015). Therefore, it is critical to identify effective diagnostic biomarkers for melanoma metastasis.

The immune system is a determining factor for tumor initiation, progression, and metastasis (Li et al., 2017). The complex interaction between the immunity and cancer cells can inhibit and promote tumor growth, and cancer immune evasion is recognized as an emerging hallmark of cancer (Hanahan and Weinberg, 2011). Melanoma is an immunogenic cancer that overcomes the control of the immune system by producing tolerable cytokines and growth factors in the microenvironment (Tucci et al., 2014). Moreover, the molecular characteristics describing tumor-immune interaction were needed to be comprehensively explored regarding their diagnostic potential in melanoma metastasis. It is important to identify a potentially reliable immune signature for melanoma metastasis because the development of melanoma is a dynamic process. Several studies have proposed gene expression-based signatures for the prognosis of patients with melanoma (Chen et al., 2017; Yang et al., 2018; Cursons et al., 2019). However, few studies provided insight into the metastasis risk.

The Cancer Genome Atlas, a large-scale public data platform, provides sequencing data for comprehensively understanding the melanoma. Several studies have drawn data of melanoma from the TCGA to identify potential prognostic biomarkers (Chen et al., 2017; Jayawardana et al., 2016; Jiang et al., 2016). The melanoma cohort in the TCGA database contains primary and metastatic samples. Specifically, TCGA did not always adopt the initially diagnosed melanoma samples for sequencing (Xiong et al., 2019). The majority of the primary samples submitted to sequence were initially diagnosed melanoma samples, and the majority of the metastatic samples for sequencing were from follow-up patients instead of initially diagnosed samples. In the present study, we used the melanoma cohort to construct the IRS to predict risk for melanoma metastasis and further selected the patients who submitted specimens on the same date as the initial diagnosis for validation.

## Materials and Methods

### Patients and Datasets

The study used data from the public domain. The cohort of melanoma for identifying immune biomarkers consists of 470 patients in TCGA. The list of IRGs was downloaded from the ImmPort database,^1^ containing a total of 1534 IRGs (Bhattacharya et al., 2014) (Supplementary Table S1). According to the list of IRGs, level 3 data of IRGs expression profiles of patients were downloaded from the TCGA database, measured experimentally using the Illumina HiSeq 2000 RNA Sequencing platform (October 13, 2017). The expression was RNA-Seq HTSeq FPKM data, which has been normalized. The project ID is TCGA-Melanoma (SKCM) and the study accession number of the TCGA data is TCGA-SKCM.htseq_fpkm.tsv (07-20-2019). In addition, the patients’ clinical information was obtained from the TCGA, including age, gender, body mass index (BMI), radiation therapy, primary melanoma sites, TNM stages, Breslow depth, and ulceration indicator (Supplementary Table S2). Because the data were extracted from the TCGA database, following the publication guidelines strictly approved by TCGA, there was no requirement for ethics committee approval.

### Training Cohort and Test Cohort

The 470 samples from 470 melanoma patients for sequencing included 103 primary samples and 367 metastatic samples. However, TCGA always took the samples from follow-up melanoma patients for sequencing. Therefore, we considered the sequencing samples as the training cohort to construct the IRS. Among 470 samples, 110 initially diagnosed samples were taken for sequencing, indicating that these 110 patients were initially diagnosed and submitted specimens on the same date. Therefore, 110 initial diagnosis patients were selected as the test cohort for validation (Supplementary Table S3), including 65 “metastasis free” patients and 45 “metastasis positive” patients. The analytic flowcharts of the study are shown in Supplementary Figure S1.

### Construction of the IRS

First, the significantly differentially expressed IRGs between primary samples and metastatic samples were first identified (fold change > 2 or < 0.5, *P* value < 0.05, and FDR < 0.05). In order to ensure the reliability of detection, the gene expression equaling to 0 in more than 50% of all samples were removed. To understand characteristic mechanisms between these genes, we performed the KEGG analysis by the “clusterProfiler” package in R software.

Then, the univariate logistic analysis was performed to identify the IRGs associated with melanoma metastasis risk (*P* value < 0.05). In the study, the concept of “melanoma metastasis risk” is the possibility of melanoma with lymph node or distant metastases. It is a binary variable (Yes or No). Last, multivariate logistic analysis was performed on these metastasis-risk related IRGs to identify the independent melanoma metastasis risk biomarkers (*P* value < 0.05). The independent metastasis risk IRGs were built the risk score model. Beta (β) coefficients of the IRGs in the multivariate logistic analysis were used as weights and to calculate the IRS: $i\,m\,m\,u\,n\,e\,r\,i\,s\,k\,s\,c\,o\,r\,e=\sum_{i=1}^{N}\left(E\,x\,p\,i\times \beta \,i\right)$. *N*, *Expi*, and β*i* represented the number of genes in IRS, gene expression level, and coefficient value, respectively.

### Performance Assessment

The performance of the predictive capability of the IRS for melanoma metastasis risk was assessed by performing the receiver operating characteristic (ROC) curve and the precision-recall curve (PR curve) and quantified by the area under the ROC curve and PR curve (AUC). According to Youden’s index, we selected the best threshold of IRS. In addition, sensitivity, specificity, negative predictive value, positive predictive value, positive likelihood ratio, and negative likelihood ratio were obtained. Besides, the clinical usefulness of the IRS was determined by quantifying the net benefits under different threshold probabilities using decision curve analysis (Vickers et al., 2008).

### The Association Between IRS and Melanoma Metastasis Risk

According to the best threshold of IRS, we divided sequencing samples into low-IRS and high-IRS groups. To estimate the association between IRS and melanoma metastasis risk, age- and multivariable-adjusted logistic regression models were performed. In the multivariable regression models, we adjusted for clinical information, such as age, gender, BMI, and others. Besides, to provide a quantitative tool of predicting the individual probability of melanoma metastasis risk, we built a diagnostic nomogram on the basis of the IRS and clinical information. Calibration curves were also plotted to compare the predicted and actual probabilities.

For validation, initial diagnosis patients were also divided into two groups using the same threshold of IRS. Then, age- and multivariable-adjusted logistic regression models, and a diagnostic nomogram were also performed.

### The Validation of IRS Using GEO Datasets

To confirm the reliability and validity of the IRS, we used other melanoma cohorts from GEO databases and normalized matrix files were downloaded directly according to the following inclusion criteria: (1) diagnosis of patients with melanoma and (2) the data from expression profiling by the array, and the following exclusion criteria: (1) the datasets with small sample sizes (*n* < 50), (2) the datasets without primary melanoma or metastatic melanoma, and (3) datasets used cell line or animal samples. At last, we selected GSE8401, GSE15605, and GSE46517 to validate the results from the TCGA database.

### Statistical Analysis

All data were expressed as mean ± SD (standard deviation). The differentially expressed IRGs were obtained using the “limma” package in software R. The AUC of ROC was estimated using the “ROCR” package. The decision curve analysis was plotted with the “rmda” package. Nomograms and calibration plots were done with the “rms” package. The above analysis was conducted using R software 3.5 and Statistical Analysis System software (SAS 9.4), and the code of the present study can be found in https://figshare.com/articles/melanoma_metastasis_code/11887548. All statistical tests were two-sided and *P* < 0.05 was considered statistically significant.

## Results

### Patient Characteristics

A total of 470 samples were included in the study, consisting of 103 primary samples and 367 metastatic samples. In the melanoma cohort, 110 samples submitted to TCGA were samples that were initially melanoma diagnosed. Therefore, 110 initial diagnosis patients were considered as test cohort, including 65 patients with Stage I or II and 45 patients with Stage III or IV. Detailed patient characteristics were given in Supplementary Table S3.

### Construction of the IRS

**FIGURE 1:**
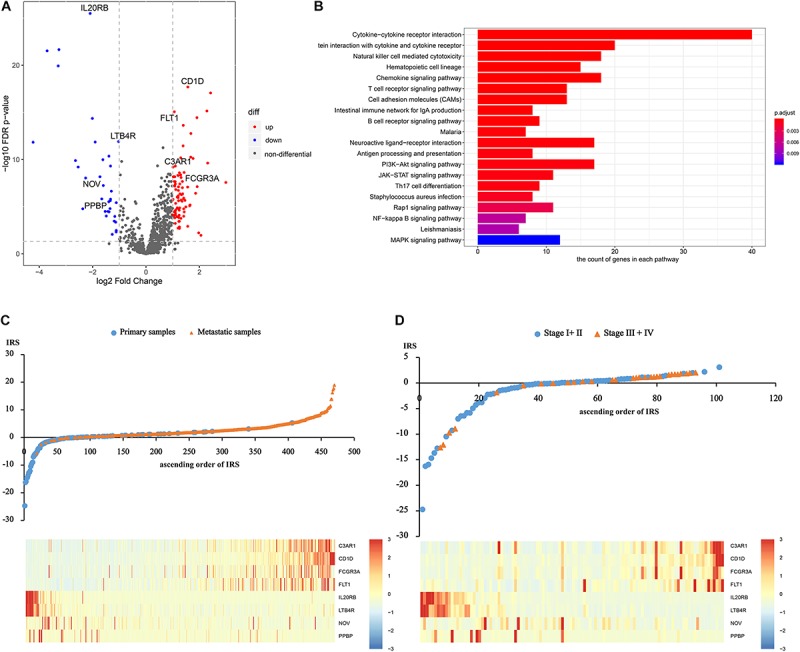
Construction of the immune risk score. **(A)** The volcano plot of significantly differentially expressed IRGs. **(B)** The significantly differentially expressed IRGs involved KEGG pathways. **(C)** and **(D)** Distribution of the IRS and gene expression among sequencing samples and the initial patients.

After differentially expressed IRGs analysis, 124 eligible IRGs were significantly differentially expressed (Supplementary Table S4), as shown in the volcano plot (Figure 1A). The KEGG analysis showed that these 124 genes were involved in melanoma metastasis by 38 KEGG pathways, including immunity and cancer-related pathways (Supplementary Table S5 and Figure 1B).

Afterward, based on the univariate and multivariate logistic analysis, only eight IRGs had a significant diagnostic value for melanoma metastasis risk (Supplementary Tables S6, S7). We calculated IRS based on the β coefficients as follows: IRS = Exp _C__3__AR__1_^∗^0.2193 + Exp _CD__1__D_^∗^1.3537 + Exp _FCGR__3__A_^∗^−0.0339 + Exp _FLT__1_^∗^0.3464 + Exp _IL__20__RB_^∗^−0.4672 + Exp _LTB__4__R_^∗^−0.1884 + Exp _*NOV*_^∗^−0.0035 + Exp _PPBP_^∗^−0.4763. The distribution of the IRS and gene expression among sequencing samples and the initial patients are given in Figures 1C,D.

### Performance Assessment

To evaluate the ability of the IRS in differentiating melanoma metastasis, the AUC of ROC was estimated. Supplementary Figure S2 shows the ROC curve and the PR curve of the IRS. As shown in Table 1, the AUC of IRS for melanoma metastasis in sequencing samples was 0.90 (95% CI = 0.86–0.93), and in initial diagnosis patients, it was 0.80 (95% CI = 0.71–0.89). Moreover, the predictive capability of the IRS was higher relative to any other clinical information (AUC < 0.70 for all) in both training and validation cohorts.

**TABLE 1:** Predictive ability of immune risk score models for melanoma metastasis.

	**Sequencing samples**		**Initial diagnosis patients**	
	**AUC**	**95% CI**	**AUC**	**95% CI**
Age	0.65	0.59–0.71	0.68	0.57–0.78
Gender	0.52	0.45–0.58	0.54	0.43–0.65
BMI	0.60	0.53–0.67	0.55	0.43–0.67
Radiation therapy	0.56	0.50–0.62		
Primary melanomas sites	0.56	0.49–0.63	0.51	0.36–0.66
Breslow depth			0.61	0.45–0.77
Ulceration indicator			0.52	0.36–0.68
Immune risk score	0.90	0.86–0.93	0.80	0.71–0.89

According to Youden’s index, we selected the best threshold (IRS = 0.60). Then, the sensitivity, specificity, negative predictive value, positive predictive value, positive likelihood ratio, and negative likelihood ratio in the sequencing samples and initial diagnosis patients were obtained (Supplementary Table S8), indicating that the IRS was reliable.

**FIGURE 2:**
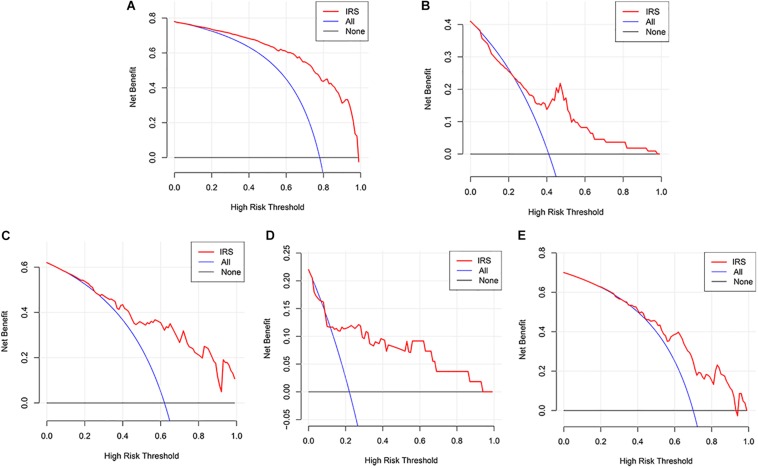
Decision curve analyses of the IRS. **(A)** Decision curve analyses for the sequencing samples. **(B)** Decision curve analyses for the initial diagnosis patients. **(C–E)** Decision curve analyses for melanoma cohorts from GEO datasets (**C:** GSE8401, **D:** GSE15605, **E:** GSE46517).

The decision curve analysis for IRS is presented in Figures 2A,B, showing that using the IRS to predict melanoma metastasis added more benefit than either all metastasis or no metastasis.

### The Association Between IRS and Melanoma Metastasis Risk

We then divided the sequencing samples and initial diagnosis patients into low-IRS and high-IRS groups using the same threshold (IRS = 0.60). To compare the IRS across the two groups for melanoma metastasis, age- and multivariable-adjusted logistic regression models were created. In age-adjusted models, patients in the group with high IRS were 18.45 times more likely to have melanoma metastasis than patients in the group with low IRS (95% CI = 10.59–32.14) in the sequencing samples. A similar increase in metastasis risk was observed in initial diagnosis patients, with the OR value of 8.93 for the high IRS compared to the low IRS (95% CI = 3.53–22.61) (Table 2). Compared with age-adjusted ORs, multivariable-adjusted ORs were attenuated but remained significant in both sequencing samples (OR = 16.35, 95% CI = 8.74–30.59) and initial diagnosis patients (OR = 7.32, 95% CI = 2.40–22.33) (Table 2).

**TABLE 2:** Age and multivariable (MV)-adjusted odds ratios for the association between immune risk score and melanoma metastasis.

	**Age-adjusted**		**MV-adjusted**	
	**OR (95%)**	***P* value**	**OR (95%)**	***P* value**
**Sequencing samples^a^**				
Low	1.00		1.00	
High	18.45 (10.59–32.14)	<0.01	16.35 (8.74–30.59)	<0.01
**Initial diagnosis patients^b^**				
Low	1.00		1.00	
High	8.93 (3.53–22.61)	<0.01	7.32 (2.40–22.33)	<0.01

*^*a*^MV-adjusted for age (continuous), gender (female, male), body mass index (BMI, <18.5, 18.5–24.9, ≥25), radiation therapy (yes, no), and primary melanoma sites (extremities/head and neck, trunk). ^*b*^MV-adjusted for age (continuous), gender (female, male), body mass index (BMI, <18.5, 18.5–24.9, ≥ 25), primary melanoma sites (extremities/head and neck, trunk), Breslow depth (continuous), and ulceration indicator (yes, no).*

**FIGURE 3:**
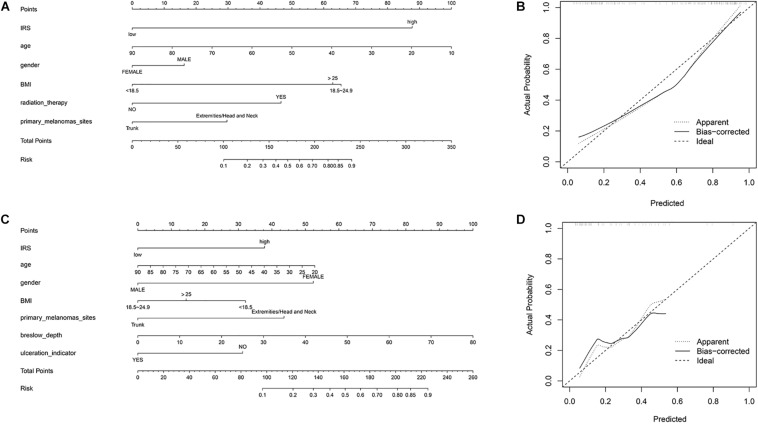
Construction of nomogram. **(A)** Nomogram for predicting melanoma metastasis for the sequencing samples. **(B)** Calibration curves of nomograms in terms of agreement between predicted and observed in the sequencing samples. **(C)** Nomogram for predicting melanoma metastasis for the initial diagnosis patients. **(D)** Calibration curves of nomograms in terms of agreement between predicted and observed in the initial diagnosis patients.

To provide a quantitative tool to predict the individual probability of melanoma metastasis risk, we constructed the diagnostic nomogram on the basis of IRS and clinical information using the sequencing samples and initial diagnosis patients, respectively (Figures 3A,C). Moreover, the calibration curve of the diagnostic nomogram demonstrated good agreement between prediction and observation in both cohorts (Figures 3B,D).

### The Validation of IRS Using GEO Datasets

Three GEO datasets including GSE8401, GSE15605, and GSE46517 were obtained to validate the IRS. We evaluated the predictive ability of IRS for melanoma metastasis risk using these datasets, showing a high accuracy for diagnosis (AUC = 0.83, 0.80, and 0.76, respectively, Table 3). In addition, we estimated the OR value by univariate logistic regression analysis. As Table 3 shows, the ORs (95% CI) were 1.09 (1.04–1.13), 1.72 (1.23–2.43), and 1.04 (1.02–1.07) in GSE8401, GSE15605, and GSE46517 datasets, respectively (Table 3), indicating that IRS was the risk factor for melanoma metastasis. The decision curve analysis also showed that using the IRS could add benefit to the diagnosis of melanoma metastasis (Figures 2C–E).

**TABLE 3:** The validation of the immune risk score in GEO datasets.

**GEO datasets**	**Primary**	**Metastasis**	**AUC**	**OR (95% CI)**	***P* value**
GSE8401	31	52	0.83	1.09 (1.04–1.13)	<0.01
GSE15605	46	12	0.80	1.72 (1.23–2.43)	<0.01
GSE46517	31	73	0.76	1.04 (1.02–1.07)	<0.01

## Discussion

Melanoma with increasing incidence and mortality rates is a public problem, attracting positive attention worldwide. If melanoma spreads through the dermis and migrants to regional lymph nodes or distant organs, the prognosis will be poor with high mortality rates (Bohme and Bosserhoff, 2016). Nowadays, many studies have built a prognostic signature to predict melanoma patients’ survival. Yang et al. (2018) identified a six-long non-coding RNA (lncRNA) signature to predict the prognosis of melanoma patients. Guo et al. (2019) mined the TCGA database to build a four-DNA methylation signature that was significantly associated with the prognosis. The expression of genes in melanoma samples was quantified by RT-PCR, and then Brunner et al. (2013) identified a nine-gene signature associated with overall survival. However, effective diagnostic biomarkers for predicting the risk of melanoma metastasis are still lacking. Therefore, we investigated the association between immune genes and melanoma metastasis to build the IRS as the metastasis predictors.

We first identified the metastasis-related genes comparing the gene expression of metastatic samples to that of primary samples. Then, the IRS consisting of eight immune genes was constructed according to the results of the univariate and multivariate logistic analysis. Among the eight immune genes in the IRS, several were reported to be significantly associated with skin or melanoma. Increased expression of FLT1, a member of the vascular endothelial growth factor receptor (VEGFR) family, is associated with glomeruloid microvascular proliferation in malignant melanoma (Straume and Akslen, 2003). RNA-seq of different tissue samples was performed to determine tissue specificity of IL20RB and LTB4R, because skin tissues had high-level expression of IL20RB and LTB4R (Fagerberg et al., 2014). As a member of the CCN family of regulatory proteins, NOV is abnormally expressed in metastatic melanoma (Vallacchi et al., 2008). The other genes were also tumor-related genes, playing an important role in various cancers. Milioli et al. (2017) found that C3AR1 was overexpressed in Basal I of breast cancer, playing a key role in cancer differentiation. CD1D encodes a divergent member of the CD1 family of transmembrane glycoproteins. Tumors can escape from the immunotherapy based on natural killer T cells by altering CD1D expression and its antigen presentation pathway (Shyanti et al., 2017). The team of Gavin (Gavin et al., 2017) reported that FCGR3A polymorphisms could predict the trastuzumab efficacy of patients with early ERBB2/HER2-positive breast cancer. Kinouchi et al. (2017) found that CXCL7 was a potential biomarker for the diagnosis of renal cell carcinoma. To justify the clinical usefulness, we further assessed whether the IRS would have power ability for predicting the risk of melanoma metastasis. The high AUC value in both sequencing samples and initial diagnosis patients demonstrated that the IRS was an effective predictor for melanoma metastasis, indicating that the immune system participated in tumor development. In addition, the decision curve analysis showed that IRS could add benefits of diagnosis.

According to the best threshold (IRS = 0.60), the sequencing samples and initial diagnosis patients were divided into two groups (high IRS and low IRS), respectively. For sequencing samples, we observed that the odds of patients with high IRS were over 18 times greater than those with low IRS in age-adjusted models. In our analysis, additionally adjusting for other clinical information did not change the results materially, indicating that the association is not solely explained by confounding by other variables. Similar results were obtained in age-adjusted models for the initial diagnosis patients. Multivariable-adjusting also did not change the results materially, suggesting that IRS might be an independent risk factor for melanoma metastasis. To improve the accuracy of predicting metastasis, we recommended that the nomogram integrate IRS, age, gender, BMI, primary melanomas sites, and other clinical information. The nomogram took into account markers from different aspects, including the immune system, basic characteristics, and indicators of melanoma, which could be a promising approach to change clinical management (Birkhahn et al., 2007). Many scholars have used this method to provide a quantitative tool for diagnosis. Huang et al. (2016) developed a radiomics nomogram with radiomics signature, CT-reported results, and clinical risk factors to predict lymph node metastasis among colorectal cancer patients. Boutros et al. (2015) built a preoperative nomogram to evaluate metastasis risk with high accuracy in primary breast cancer. Silva et al. (2015) also provided an easy and practical nomogram to estimate the risk of prostate cancer. Moreover, the calibration curve showed that the nomogram with IRS could predict the melanoma metastasis accurately in both sequencing samples and initial diagnosis patients.

Nowadays, several studies have been committed to finding the signature of melanoma metastasis. Cadili et al. (2010) considered the 2 and 5 mm for total sentinel lymph node metastasis as the cutoff value, which could effectively predict the non-sentinel lymph node metastasis in melanoma patients. Wardwell-Ozgo et al. (2014) demonstrated that homeobox transcription factor A1 (HOXA1) mediated the cell invasion in melanoma cells, and primary tumors with high-expression HOXA1 were high-risk metastasis subgroups. Ferretti et al. (2016) found that BMI1 could be able to identify primary tumors that were likely to become metastatic, which was a key determinant of melanoma metastasis. The present study differed from previous reports about melanoma metastasis and had its own advantages. First, no studies provided an immune signature for melanoma metastasis. Because melanoma is an immunogenic cancer, immune-related biomarkers with multiple genes may be more effective than others. Second, we used the age- and multivariable-adjusted logistic regression models to estimate the odds of IRS in samples, which could explain that the IRS was the independent risk factor for melanoma metastasis. Third, we built the IRS using sequencing samples and validated it in the initial diagnosis patients. Moreover, we further confirmed its power diagnostic ability in three independent melanoma cohorts from the GEO database, avoiding overfitting of the IRS model.

Nevertheless, the study remains a few limitations. First, some risk factors for melanoma, such as ultraviolet radiation (El Ghissassi et al., 2009), pigmentary (Veierod et al., 2010), family history of melanoma (Watts et al., 2017), and others, were not collected in the TCGA and GEO database. In the future, we further comprehensively verified the model in other melanoma cohorts. Another limitation is potential reporting bias because all samples were from the retrospective collection. Further prospective studies are required to validate the results. Taken together, the study provided comprehensive insights into the immune microenvironment of melanoma and was the first to identify the immune signature to predict the melanoma metastasis risk.

## Data Availability Statement

Publicly available datasets were analyzed in this study. These data can be found in the TCGA database (https://tcga-data.nci.nih.gov/tcga/).

## Author Contributions

YS, LY, and LG conceived and designed the study. YS and CY performed the bioinformatic analysis and wrote the manuscript. LT and LN contributed to the revision of the manuscript draft. All authors read and approved the final manuscript.

## Conflict of Interest

The authors declare that the research was conducted in the absence of any commercial or financial relationships that could be construed as a potential conflict of interest.

## Footnotes

1 https://www.immport.org/shared/geneData/GOappend1.xls

## Supplementary Material

FIGURE S1 The analytic flowcharts of the study.

FIGURE S2 The ROC curve and the PR curve of the IRS. **(A)** The ROC curve of the IRS in the sequencing samples. **(B)** The PR curve of the IRS in the sequencing samples. **(C)** The ROC curve of the IRS in the initial diagnosis patients. **(D)** The PR curve of the IRS in the initial diagnosis patients.

TABLE S1 The list of immune genes.

TABLE S2 The information of samples from the TCGA.

TABLE S3 Samples’ basic characteristics.

TABLE S4 The significantly differentially expressed genes.

TABLE S5 The KEGG analysis.

TABLE S6 The results of univariate logistic analysis.

TABLE S7 Coefficients of immune genes in the multivariate logistic analysis.

TABLE S8 The summary statistics for IRS.

## Abbreviations

95% CI: 95% confidence interval
AUC: area under the curve
GEO: Gene Expression Omnibus
IRGs: immune-related genes
IRS: immune risk score
KEGG: Kyoto Encyclopedia of Genes and Genomes
OR: odds ratio
ROC: receiver operating characteristic
SD: standard deviation
TCGA: the Cancer Genome Atlas.
