# Supplementary figures and images for: Predicting the Risk of Melanoma Metastasis Using an Immune Risk Score in the Melanoma Cohort
Source: Front Bioeng Biotechnol. 2020 Mar 31;8:206. doi: 10.3389/fbioe.2020.00206 (PMC7136491; doi:10.3389/fbioe.2020.00206)

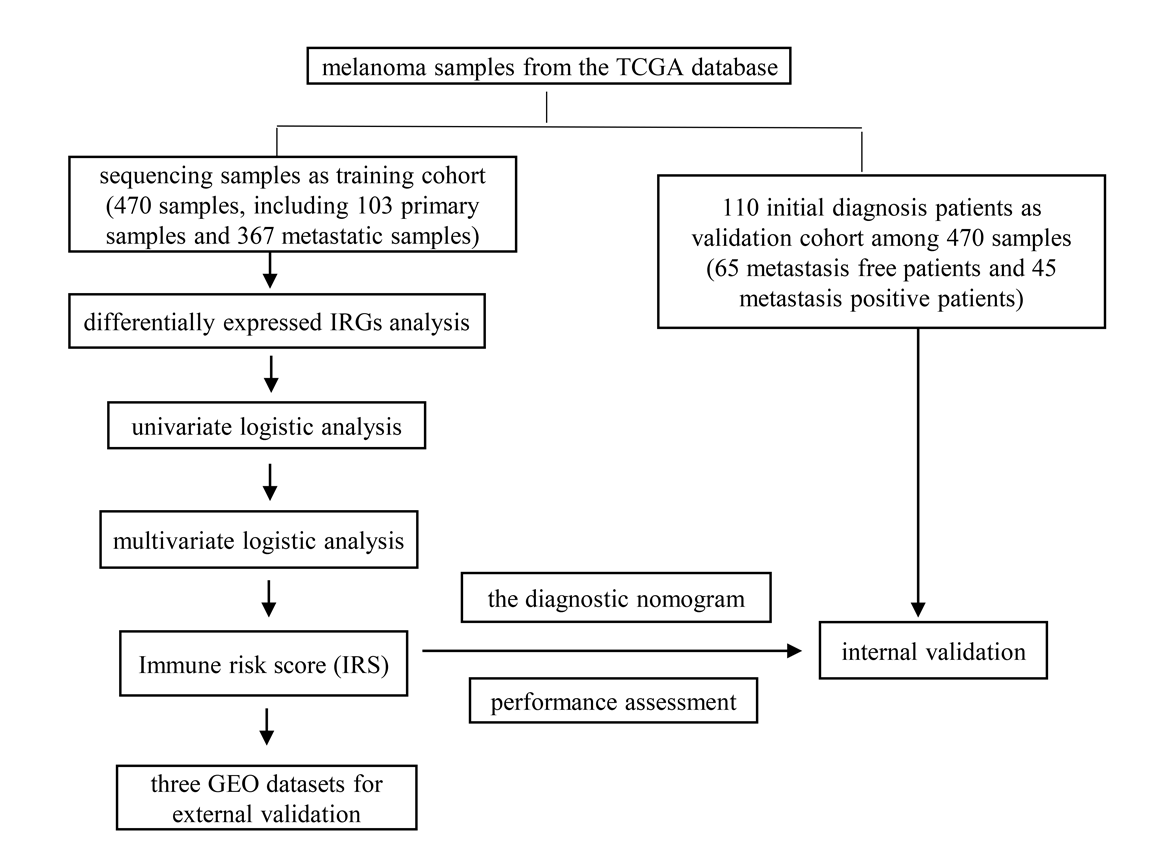

Supplement: FIGURE S1 — The analytic flowcharts of the study. [file Image_1.TIF]

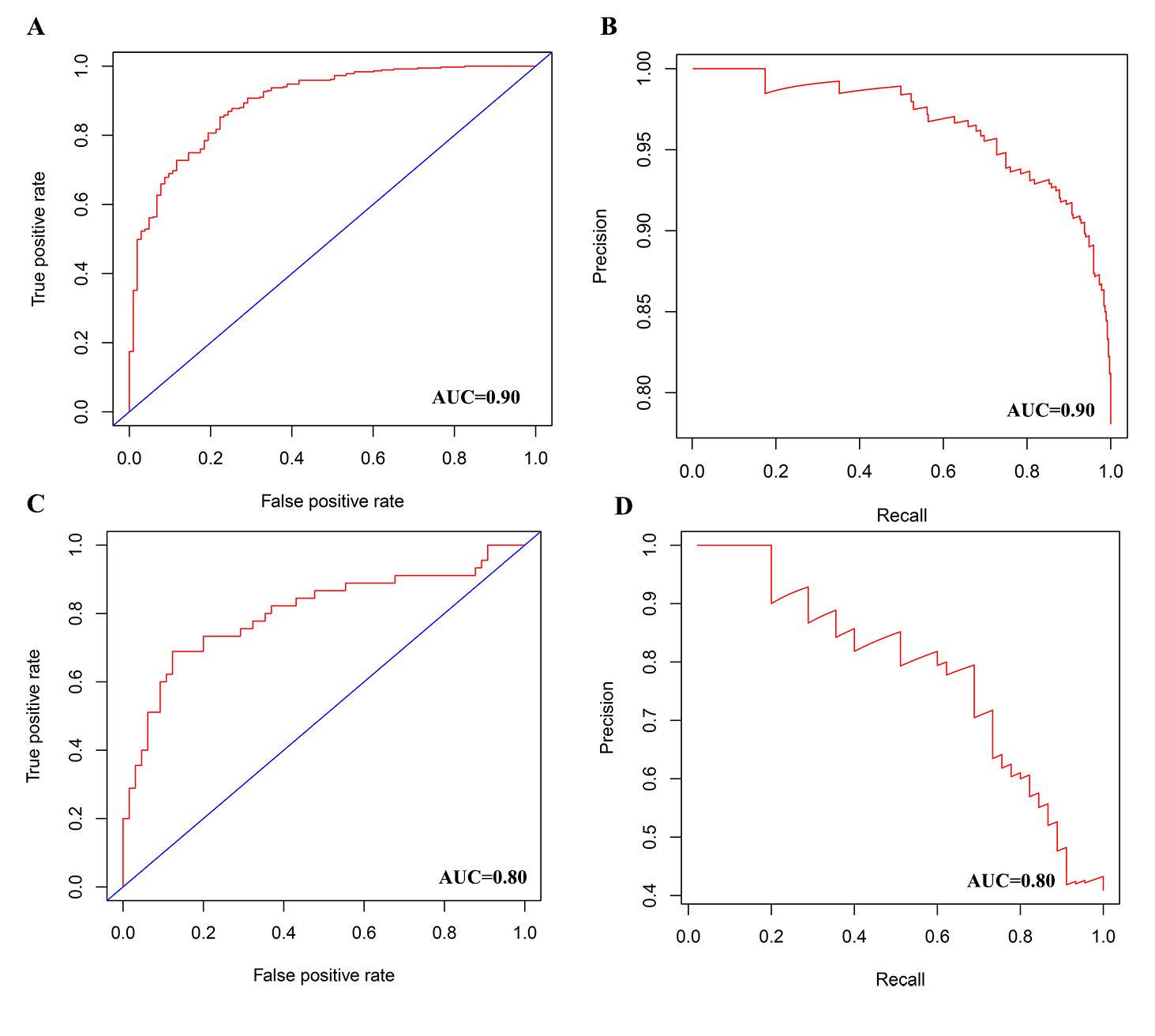

Supplement: FIGURE S2 — The ROC curve and the PR curve of the IRS. (A) The ROC curve of the IRS in the sequencing samples. (B) The PR curve of the IRS in the sequencing samples. (C) The ROC curve of the IRS in the initial diagnosis patients. (D) The PR curve of the IRS in the initial diagnosis patients. [file Image_2.TIF]
