# Supplementary material for: Predicting the Risk of Melanoma Metastasis Using an Immune Risk Score in the Melanoma Cohort
Source: Front Bioeng Biotechnol. 2020 Mar 31;8:206. doi: 10.3389/fbioe.2020.00206 (PMC7136491; doi:10.3389/fbioe.2020.00206)
Supplement: TABLE S3 — Samples’ basic characteristics. [file Table_3.DOCX]

|  | **Sequencing sample type (n=470)** | | **Initial diagnosis patients (n=110)** | |
| --- | --- | --- | --- | --- |
|  | **Primary samples** | **Metastatic samples** | **metastasis free** | **metastasis positive** |
| **N** | 103 | 367 | 65 | 45 |
| **Age** | 64.71±13.89 | 56.30±15.70 | 65.98±12.43 | 56.20±16.90 |
| **Gender** |  |  |  |  |
| male | 61 | 137 | 38 | 23 |
| female | 42 | 230 | 27 | 22 |
| **BMI** |  |  |  |  |
| <18.5 | 5 | 4 | 2 | 2 |
| 18.5～24.9 | 32 | 42 | 22 | 9 |
| ≥25 | 53 | 114 | 37 | 24 |
| **Radiation therapy** |  |  |  |  |
| yes | 2 | 48 | 1 | 6 |
| no | 101 | 318 | 64 | 39 |
| **Primary melanomas Sites** |  |  |  |  |
| extremities/ head and neck | 40 | 143 | 26 | 10 |
| trunk | 39 | 90 | 24 | 9 |
| **Breslow depth (mm)** | 11.73±12.73 | 3.52±4.70 | 9.91±10.13 | 17.65±20.38 |
| **Ulceration indicator** |  |  |  |  |
| yes | 76 | 91 | 44 | 14 |
| no | 13 | 133 | 10 | 4 |

**Supplementary table 3.** **samples’ basic characteristics**
