# Supplementary material for: Predicting the Risk of Melanoma Metastasis Using an Immune Risk Score in the Melanoma Cohort
Source: Front Bioeng Biotechnol. 2020 Mar 31;8:206. doi: 10.3389/fbioe.2020.00206 (PMC7136491; doi:10.3389/fbioe.2020.00206)
Supplement: TABLE S7 — Coefficients of immune genes in the multivariate logistic analysis. [file Table_7.DOCX]

| Symbol | β | OR | 95% CI | P value |
| --- | --- | --- | --- | --- |
| C3AR1 | 0.2193 | 1.245 | 1.081-1.434 | 0.0024 |
| CD1D | 1.3537 | 3.872 | 1.682-8.911 | 0.0015 |
| FCGR3A | -0.0339 | 0.967 | 0.944-0.989 | 0.006 |
| FLT1 | 0.3464 | 1.414 | 1.099-1.819 | 0.007 |
| IL20RB | -0.4672 | 0.627 | 0.478-0.822 | 0.0007 |
| LTB4R | -0.1884 | 0.828 | 0.700-0.981 | 0.0288 |
| NOV | -0.00351 | 0.996 | 0.994-0.999 | 0.0185 |
| PPBP | -0.4763 | 0.621 | 0.466-0.827 | 0.0011 |

**Supplementary table 7. Coefficients of immune genes in the multivariate logistic analysis**
