# Supplementary material for: Predicting the Risk of Melanoma Metastasis Using an Immune Risk Score in the Melanoma Cohort
Source: Front Bioeng Biotechnol. 2020 Mar 31;8:206. doi: 10.3389/fbioe.2020.00206 (PMC7136491; doi:10.3389/fbioe.2020.00206)
Supplement: TABLE S8 — The summary statistics for IRS. [file Table_8.DOCX]

|  | Sequencing samples | Initial diagnosis patients |
| --- | --- | --- |
| Cut-off of IRS | 0.60 | 0.60 |
| accuracy | 0.84 | 0.75 |
| Sensitivity | 0.85 | 0.69 |
| Specificity | 0.78 | 0.8 |
| Positive predictive value | 0.93 | 0.7 |
| Negative predictive value | 0.6 | 0.79 |
| Positive likelihood ratio | 3.82 | 3.44 |
| Negative likelihood ratio | 0.19 | 0.39 |

**Supplementary table 8. The summary statistics for IRS**
